# Impact of neoadjuvant treatment on total mesorectal excision for ultra-low rectal cancers

**DOI:** 10.1186/1477-7819-8-23

**Published:** 2010-03-26

**Authors:** Yon Kuei Lim, Wai Lun Law, Rico Liu, Jensen TC Poon, Joe FM Fan, Oswens SH Lo

**Affiliations:** 1 Department of Surgery, The University of Hong Kong, Queen Mary Hospital, Hong Kong; 2 Department of Clinical Oncology, Queen Mary Hospital, Hong Kong

## Abstract

**Background:**

This study reviewed the impact of pre-operative chemoradiotherapy or post-operative chemotherapy and/or radiotherapy on total mesorectal excision (TME) for ultralow rectal cancers that required either low anterior resection with peranal coloanal anastomosis or abdomino-perineal resection (APR). We examined surgical complications, local recurrence and survival.

**Methods:**

Of the 1270 patients who underwent radical resection for rectal cancer from 1994 till 2007, 180 with tumors within 4 cm with either peranal coloanal anastomosis or APR were analyzed. Patients were compared in groups that had surgery only (Group A), pre-operative chemoradiotherapy (Group B), and post-operative therapy (Group C).

**Results:**

There were 115 males and the mean age was 65.43 years (range 30-89). APR was performed in 134 patients while 46 had a sphincter-preserving resection with peranal coloanal anastomosis. The mean follow-up period was 52.98 months (range: 0.57 to 178.9). There were 69, 58 and 53 patients in Groups A, B, and C, respectively. Nine patients in Group B could go on to have sphincter-saving rectal resection. The overall peri-operative complication rate was 43.4% in Group A vs. 29.3% in Group B vs. 39.6% in Group C, respectively. The local recurrence rate was significantly lower in Group B (8.6.9% vs. 21.7% in Group A vs. 33.9% in Group C) *p < 0.05*. The 5-year cancer-specific survival rates for Group A was 49.3%, Group B was 69.9% and Group C was 38.8% (*p *= 0.14).

**Conclusion:**

Pre-operative chemoradiation in low rectal cancer is not associated with a higher incidence of peri-operative complications and its benefits may include reduction local recurrence.

## Background

In rectal cancer surgery, resection of the tumor and draining lymph nodes with adequate distal and circumferential margins is the most important aim. Tumors in the distal rectum have always been a challenge in their management in terms of a higher local recurrence rate, when compared to upper or mid rectal cancers [1,2]. Very distal rectal cancers, especially those involving the anal sphincters are mostly treated surgically with an abdomino-perineal resection (APR). In resectable rectal adenocarcinoma, improved prognosis has been attributed to advances in surgical technique, namely total mesorectal excision (TME), which is now the gold-standard procedure for mid and distal rectal cancer, with local recurrence rates of less than 10 per cent [3-6].

With advances in the surgical techniques, better understanding of disease spread, and the appropriate use of post-operative chemo/radiotherapy, some of the distal cancers can now be resected with restoration of intestinal continuity, without compromising local recurrence rates at the same time[7,8]. APR is now reserved only for distal rectal cancer when an anastomosis is not possible.

Furthermore, randomized studies have shown benefits of post-operative chemo/radiotherapy therapy over surgery alone [9-11] in the presence of optimal surgery by TME. Radiotherapy can either be given preoperatively or postoperatively. The advantages of neoadjuvant therapy over postoperative adjuvant therapy include: better local control of disease, reduced therapeutic toxicity and increasing the possibility of sphincter preservation [12-16]. However, there are concerns of neoadjuvant therapy on the early post-operative morbidity [17,18].

The objective of this study was to examine the impact of multimodality treatment of either pre-operative chemoradiation or post-operative therapy on peri-operative morbidity and the oncological outcomes of patients who underwent TME surgery specifically for very low rectal cancers that required either low anterior resection with per anal coloanal anastomosis or abdomino-perineal resection (APR) from a single specialized tertiary Asian institution.

## Methods

### Patient population

From 1994 till 2007, 1270 patients underwent radical resection for rectal cancer in the Department of Surgery, The University of Hong Kong, Queen Mary Hospital. Among them, 180 consecutive patients who suffered from very low rectal adenocarcinoma with the tumor within 4 cm from the anal verge and underwent TME with either abdomino-perineal resection or ultra-low anterior resection (ULAR) with hand-sewn coloanal anastomosis were included in this study. Data on the patients' demographics, operative details, postoperative outcome and follow-up status were collected in a prospective database for colorectal malignancy. They either underwent surgery alone (Group A), or had preoperative chemoradiotherapy before surgery (Group B), or underwent postoperative adjuvant therapy (Group C). Patients with pathologies other than adenocarcinoma, those undergoing emergency surgery, and those with previous rectal surgery were excluded.

The management protocol was described in our previous publications [19,20]. All the patients underwent colonoscopy with biopsy-proven adenocarcinoma of the rectum. Pre-operative clinical staging comprised of a combination of physical and per rectal examination, with various imaging modalities (Computed Tomography scan, Magnetic Resonance Imaging, endoscopic ultrasound).

### Policy of adjuvant therapy

In the early part of the study, preoperative chemoradiation was only given to those with T4 disease. In patients who underwent APR, based on the hypothesis that perineal dissection could not achieve dissection along a well-defined plane and that tumor implantation at the time of operation was more likely as well as on the poor reported oncological results of APR even using TME [21], postoperative chemoradiation was offered to patients with stage II and stage III disease in the early period. In those patients with low anterior resection with TME, post-operative radiation was not given during the initial period of the study and postoperative chemotherapy was offered to those with stage II or stage III disease.

After our analysis of the results of patients who underwent APR and low anterior resection with hand-sewn coloanal anastomosis [19], preoperative chemoradiation was offered to those who had T3 disease and/or positive lymph nodes by preoperative staging and were designated to have APR or hand-sewn coloanal anastomosis. From 2004, a combined multidisciplinary meeting with surgeons, radiologists and clinical oncologists decided the policy of adjuvant therapy of patients on individual basis. Neoadjuvant treatment comprised of long course 5-fluorouracil (5 FU) - based concurrent chemotherapy (IV bolus) and radiation (4500-5040 Gy). External beam radiation therapy was delivered at 180 cGy daily in either 3-or 4-field technique for 5 weeks, followed by 540-cGy boosts. Surgery was subsequently carried out within 4-6 weeks after completing neoadjuvant treatment. The surgical approach of open versus laparoscopic resection was chosen by the surgeon, based on the experience and technical skills.

### Surgical Technique

All patients underwent bowel preparation with polyethylene glycol electrolyte solution the day before the operation and surgery was performed in the Lloyd-Davis position. Prophylactic intravenous antibiotics were used routinely at induction of general anesthesia.

Total mesorectal excision with rectal mobilization being carried out by sharp dissection under direct vision keeping the fascia propia of the mesorectum intact. Operative techniques for APR and LAR have been previously described [19]. The decision for APR was made if the tumor invaded the anal sphincters, an inadequate distal margin, or if patient had poor sphincter function preoperatively. In patients who were suitable for a peranal coloanal anastomosis, the perineal surgeon completed the excision transanally at the dentate line via an intersphincteric dissection, whereby the plane between the internal and external sphincters was dissected proximally until it met with the intraperitoneal dissection. The Lone Star retractor (Cooper-Surgical, Trumbull, CT) was used for exposure and a hand-sewn single-layer interrupted anastomosis was performed transanally. Intestinal continuity is reestablished after completion of adjuvant therapy, following clinical and radiological evidence of anastomotic integrity.

All patients had fecal diversion with either a loop ileostomy or a loop transverse colostomy if the ileum was not suitable.

### Postoperative management

Pathological staging of patients was performed according to the postoperative pathological report by using the standard tumor node metastasis (TNM) system (AJCC 6^th ^Ed). Complete pathological response was defined as the absence of viable tumor cells in the specimen. The definition of anastomotic leakage was based on intra-operative findings on reoperation or in the case of conservatively managed patients, based on clinically directed imaging investigations.

### Follow up

Patients were followed up at 2-3 month intervals in the first 2 years, then 4-6 months until 5 years after surgery and yearly thereafter. History and physical examination were performed at each follow-up visit. Digital rectal examination was performed in all patients who had ULAR to check for anastomotic recurrence or stricture. Serum carcino-embryonic antigen (CEA) level was measured at each visit. Imaging and/or colonoscopy were performed in patients in whom recurrence was suspected. Local recurrence was defined as biopsy-proven pelvic recurrence below sacral promontory or a rise in serum CEA with positive imaging studies. Lung, liver and biopsy-proven inguinal lymphadenopathy was considered as distant metastatic disease.

### Statistical method

Proportions were compared using Chi-squared test or Fisher exact test. Continuous variables were presented as median (range) and were compared using Mann-Whitney U test when appropriate. One-way ANOVA was used to check equivalence of means of the 3 groups. Cancer-specific survival and local recurrence were analyzed by the Kaplan-Meier method; comparison made with log rank test. P values of less than *0.05 *were considered statistically significant. Confidence intervals were defined at 95 per cent.

## Results

Over 14 years, 180 patients had very low rectal cancer and required either per anal coloanal anastomosis or abdomino-perineal resection. The characteristics of the patients, operations and tumors are shown in Table 1. There were 115 males and 65 females with a mean age of 65.43 years (range 30-89). One hundred and of thirty-four of them underwent an APR while 46 had a sphincter-preserving ultra-low anterior resection with per anal coloanal anastomosis, combined with defunctioning stomas (40 ileostomies and 6 colostomies). Laparoscopic resection was used in 38 patients. All the surgeries were performed electively with no incidence of perforation or obstruction at the time of surgery. The cohort had a mean follow-up period of 52.98 months (range: 0.57 to 178.9 months). Sixty-nine patients underwent surgery only, while 58 patients received pre-operative chemoradiotherapy, and 53 patients had post-operative adjuvant therapy in addition to surgery. Nine out of the 58 patients with preoperative chemoradiotherapy could have a sphincter-saving rectal resection. In Group C, 26 patients had chemotherapy alone, 6 patients had just radiotherapy and 21 patients had concomitant chemoradiotherapy after the operation.

**Table 1:** Patient, tumor and surgical characteristics

*Characteristics*	*Group A* *(n = 69)*	*Group B* *(n = 58)*	*Group C* *(n = 53)*	*P value*
Mean(range)Age (yrs)	70.6(33-88)	63.1(30-88)	61.2(37-89)	*NS*
Gender				
Male	36	35	35	*NS*
Female	33	23	18	
Comorbidities				*NS*
ASA status				*NS*
1	11	9	13	
2	44	43	34	
3	13	6	6	
4	1	0	0	
Type of Surgery				
APR	53	49	32	*0.04*
ULAR	16	9	21	
Intent of Surgery				*NS*
Curative	62	54	49	
Palliative	7	4	4	
Approach (Lap/Open)	16/53	12/46	10/43	*NS*
Median(range)Operating time(mins)	174.5(80-340)	185.3(75-420)	181.8(100-340)	*NS*
Median(range)Blood loss intraop (mls)	601(50-3000)	842(99-4200)	654(99-2000)	*NS*
Pathological tumor stage				
1	28	8	2	
2	19	28	13	
3	16	16	34	
4	4	1	4	
Tumor differentiation				*NS*
Poor	8	8	12	
Moderate	52	38	39	
Well	5	4	1	
Neurovascular involvement	14	13	29	*NS*

ASA, American Society of Anesthesiologist, APR, abdomino-perineal resection, ULAR, ultra-low anterior resection, NS, not significant

The 3 groups were comparable in terms of demographics, age, gender, comorbidities, as well as American Society of Anesthesiology (ASA) score. The type of surgery, approach (laparoscopic vs. open) and surgery with curative intent were also similar.

There was no statistical difference in the operating time, blood loss and intra-operative complications among the patients in the three groups. Two patients in Group A had ureteric injury while there were no significant intra-operative complications in patients of the other groups.

The overall peri-operative complication rate was 43.4% in Group A, 29.3% in Group B and 39.6% in Group C (Table 2). The breakdown of various complications among the groups was not significantly different. The complications are as follows: Cardiac complications (commonest being atrial fibrillation, congestive cardiac failure, and non-fatal myocardial infarction) were 10.1% vs. 1.7% vs. 1.9% in Groups A, B, and C, respectively. Pulmonary complications (commonest were pneumonia and atelectasis) were 1.4% vs. 1.7% vs. 7.5%. Urological complication rates (urinary tract infection and acute retention of urine) were: 17.4% vs. 3.4% vs. 5.7%. As for surgical complications: the wound infection rates were 8.7% vs. 13.8% vs. 15.1%. The anastomotic leak rate as earlier defined was 0 vs. 22% vs. 10% respectively. The re-operation rates (for leak, abscess or bleeding) were 10.1% vs. 5.2% vs. 3.8%. The overall 30-day mortality was 0.56%(1 patient had small bowel gangrene from Group A).

**Table 2:** Postoperative complications

Complications	Group A n (%)	Group B n (%)	Group C n (%)	*P *value
Cardiac	7 (10.1)	1 (1.7)	1 (1.9)	*NS (0.272)*
Pulmonary	1(1.4)	1 (1.7)	4 (7.5)	*NS (0.140)*
Urinary	12 (17.4)	2 (3.4)	3 (5.7)	*NS (0.380)*
Surgical				
Intraoperative	2 (2.8)	0 (0)	1 (1.9)	
Postoperative	1 (1.4)	2 (3.4)	0 (0)	*NS (0.314)*
Wound	6 (8.7)	8 (13.8)	8 (15.1)	*NS (0.209)*
Leak	0 (0)	2 (22)	2 (10)	*NS (0.438)*
Re-operation	7 (10.1)	3 (5.2)	2 (3.8)	*NS (0.604)*
30-day mortality	1 (1.4)	0 (0)	0 (0)	*NS*
Overall perioperative complication	30 (43.4)	17 (29.3)	21 (39.6)	*NS*
				*NS*

**Figure 1:**
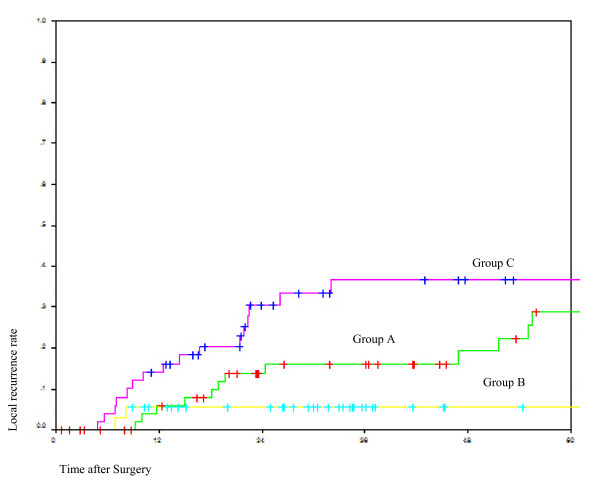
**Comparison of local recurrence among the three groups**. Green line = Group A Surgery alone; Yellow line = Group B: Preoperative chemoradiation; Red line = Group C: Postoperative therapy.

The oncological outcomes are shown in Table 3. The 5-year local recurrence rate was significantly lower in Group B (8.6% vs. 21.7% in Group A vs. 33.9% in Group C) (*p = 0.012)*(Fig 1). The median time to the occurrence of local recurrence for Group A was 53.3 months (range 0.57 - 176.3 months), Group B was 33.78 months (range 6.87 - 139.3 months), and Group C was 41.52 months(range 4.77 - 135.8 months). There was no significant difference in the systemic recurrence among the 3 groups of patients.

**Table 3:** Long-term follow up results

Recurrence	Group A n (%)	Group B n (%)	Group C n (%)	*P *value
Local Recurrence	15 (21.7)	5 (8.6)	18 (34)	0.012
Median(range) disease free(months)	53.3(0.57-176)	33.78(6.8-139)	41.5(4.7-136)	*NS*
Systemic recurrence	14 (20.3)	13(22.4)	18(33.9)	*NS *(0.543)
5 year cancer-specific survival rate	(49.3)	(69.9)	(38.8)	0. 14

**Figure 2:**
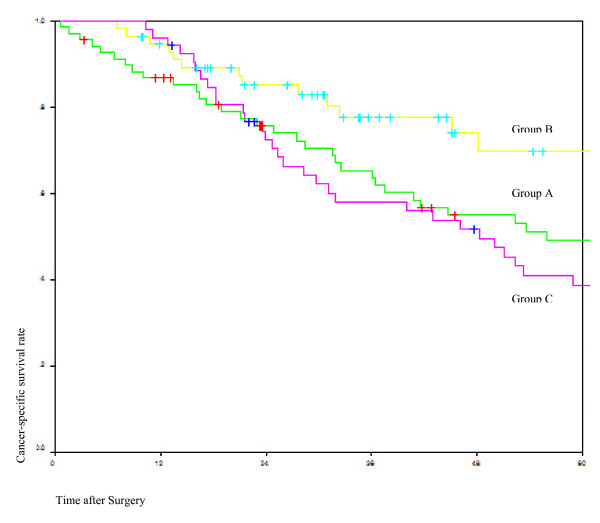
**Comparison of cancer specific survival of the three groups**. Green line = Group A Surgery alone; Yellow line = Group B: Preoperative chemoradiation; Red line = Group C: Postoperative therapy.

The 5-year survival rate for Group A was 49.3%, Group B was 69.9% and Group C was 38.8% (*p *= 0.14). (Fig 2)

## Discussion

Total mesorectal excision (TME) has been used as standard surgical treatment for rectal cancers, contributing significantly to lowered local recurrence rates [22,23]. Especially for distal rectal cancer, local recurrence is an important end-point for success because it occurs with significant frequency, is difficult to manage effectively and causes significant morbidity. Previously, our center has shown that TME with or without sphincter ablation for patients with distal rectal cancer was associated with low mortality rate and acceptable morbidity [19]. Those patients who underwent total mesorectal excision with double stapling anastomosis, the local recurrence rate was 7.1%[24]. For the subgroup of patients very low rectal cancers treated with wider surgery (APR) or low anterior resection with peranal coloanal anastomosis, the local control and survival were worse than those with double stapling anastomosis. This leads to next question of the role of adjuvant therapy for these patients. Numerous randomized trials for rectal cancer have now demonstrated the multimodality treatment can further improve local control even after optimal TME has been performed [25-28]. However, when to give chemotherapy and radiotherapy is also a point for debate [29].

Comparing preoperative against postoperative chemoradiation, the often cited theoretical advantages of preoperative therapy include a well-vascularized tumor target which would optimize tumor response rates, absence of adhesions and surgical changes which may minimize the toxicity involved with small-bowel irradiation. In addition, preoperative chemoradiation does reduce local recurrence, increase resectability of rectal cancer, improve possibility of sphincter preservation, and improve disease-free and overall survival [30-32]. Our experience for these 180 patients with distal rectal cancer did demonstrate that there was a significantly reduced incidence of local recurrence when chemoradiation was given preoperatively, regardless of whether an APR or ULAR was performed.

However, few recent studies have reported concerns of the effects of preoperative irradiation on operative time, blood loss and post-operative complication rate, namely perianal sepsis, delayed surgical wound healing, and anastomotic dehiscence [33,34]. In our series, these concerns did not appear to be significant, which is in agreement with other studies, which have found no increase in early postoperative complications with standard course preoperative chemoradiotherapy. Aggressive chemoradiation has been shown not to affect QoL and anorectal function [35].

Examining the stage of the tumors of the 3 groups, it is noted that the majority of patients in Group A had early stage 1 disease, and hence underwent upfront surgery only. In contrast, Group C comprised of more advanced disease, and there was understandably selection bias in decision for sending these patients for postoperative therapy. Despite this, Group B had significant lower local recurrence rate as well as longer 5-year survival than Group A, which could be due to the beneficial effects of preoperative therapy. However, there are limitations to assess patients in group B, as they had undergone preoperative therapy which would have downstaged the tumour.

Inherent in any retrospective analysis, limitations and bias do exist. These include difficulties in patient selection, which is a complex process especially in distal rectal cancer. Moreover, the comparative study of available data is complex because of different inclusion criteria and chemoradiotherapy modalities used as well as the policy of preoperative therapy during the study period with the analysis of the data. 35 patients with stage 2/3 did not receive pre-operative chemoradiation because they were in the pre-chemoradiation era. Although, our series is not a randomized blinded study, but given the standardized management, homogeneity of the groups and the relatively long surgical practice of our team, the results do represent a significant finding in terms of outcomes of the various groups. This also demonstrated how the change in the policy of adjuvant therapy could help to improve the results of the patients.

Most recently, there is convincing and consistent evidence that short-course preoperative radiotherapy is an effective treatment for patients with operable rectal cancer [36]. However, it may not give yield superior survival rates compared to conventional fractionated long course chemoradiotherapy [37]. Randomized studies by The Trans-Tasman Radiation Oncology Group trial (TROG 0.104) and the Stockholm III trial may provide more answers in this aspect of the best way to give neoadjuvant therapy.

In the modern context of laparoscopic TME, the current data does not appear to show that pre-operative chemoradiation treatment would jeopardize the perioperative results compared to open surgery[38]. However, randomized controlled trials may be required to shed more light in this area. Our data shows laparoscopic surgery was carried out in equal proportions in the 3 different groups analyzed.

## Conclusion

Pre-operative chemoradiation in patients with distal rectal cancer treated with surgery is not associated with a higher incidence of peri-operative complications. Its benefits may include reduction in local recurrence.

## Competing interests

The authors declare that they have no competing interests.

## Authors' contributions

YKL: analysis of the data and drafting of the manuscript. RL: input on the radiation therapy treatment and provision data on radiation therapy. JP: review of manuscript. JKMF, SHL: collection and analysis of data. WLL: study design, supervision and review of manuscript. The authors have read the manuscript and are in agreement with its contents.
